# Anti-tuberculosis drug-induced acute liver failure requiring transplantation in the second trimester of pregnancy: a case report

**DOI:** 10.1186/s12884-021-04065-0

**Published:** 2021-08-31

**Authors:** Zhoufeng Zhu, Min Zhang, Yang Li

**Affiliations:** Department of Gynecology and Obstetrics, The First Affiliated Hospital, College of Medicine, Zhejiang University, No. 79 Qingchun Road, 310003 Hangzhou City, Zhejiang Province China

**Keywords:** Anti-tuberculosis drugs, Hepatotoxicity, Pregnancy, Liver failure, Liver transplantation, Case report

## Abstract

**Background:**

Treatment of tuberculosis (TB) during pregnancy can reduce maternal and foetal complications. However, it may also induce fatal liver injury.

**Case presentation:**

We present a case of a 26-year-old pregnant woman who underwent orthotopic liver transplantation for anti-TB drug-induced fulminant hepatic failure (FHF). Her tuberculous pleurisy was treated with rifampin, isoniazid and pyrazinamide. An artificial liver support system (ALSS) was unable to reverse the liver injury while serving as a bridge to liver transplantation. She had a successful liver transplantation operation at 17 3/7 weeks of gestation. The foetal ultrasound scan showed mild foetal bilateral ventriculomegaly at 21 5/7 weeks of gestation, and labour was induced via double-balloon catheter as soon as the allograft function was stable. Despite immunosuppression, the TB was well controlled with linezolid, levofloxacin and pyridoxine at the 8 months follow-up.

**Conclusions:**

Anti-TB drug-induced liver failure during pregnancy is rare. We present a case of successful treatment of FHF in which an artificial liver support system combined with liver transplantation. The FHF was caused by anti-TB drugs with difficulties due to pregnancy status and post-transplant anti-TB treatment. Mild foetal ventriculomegaly was found in our case. Further research is still needed to identify the risks of TB treatment and liver transplantation in pregnant women. A multidisciplinary team coordinated properly to optimize patient outcomes.

## Background

Tuberculosis (TB) is a common infectious disease, and it is estimated that 216,500 pregnant women worldwide had active TB in 2013 [1]. In China, the national total TB incidence was approximately 1.41 million in 2017 [2]. Despite the large number, information on pregnancy-related TB is still inadequate. Indeed, active TB in pregnancy represents a significant problem for both women and foetuses. Timely and appropriate TB treatment is vital to prevent maternal and perinatal complications [3]. However, anti-tuberculosis drug-induced liver dysfunction is a major adverse effect. The reported incidence of standard multidrug anti-TB drug-induced liver injury (DILI) varies between 2 and 28 % according to different populations and definitions [4]. DILI may manifest with a broad spectrum of clinical features, from asymptomatic elevation of liver enzyme levels to fulminant liver failure [5]. Nevertheless, it is difficult to predict which patient will develop hepatotoxicity during tuberculosis treatment. There are only a few reports on liver transplantation (LT) for TB patients, since active TB is considered to be a relative contraindication. The risk of aggressive dissemination of the disease after transplantation has not been clearly determined for the current anti-TB regimen [6]. Michele et al. reviewed 26 cases of LT performed in patients with concomitant active TB and liver failure secondary to anti-TB treatment toxicity [7]. In these cases, only one patient, who had undetectable HIV before surgery, died due to uncontrolled TB, and another 22 patients (85 %) were alive after a median follow-up of 12 months. Many reported pregnancies with positive outcomes have been reported for women who underwent LT before the pregnancy. However, experience in liver transplantation in pregnant patients is still lacking worldwide. We present a unique case of LT in a patient in middle trimester pregnancy with concomitant tuberculous pleurisy and hepatic failure.

## Case presentation

A 26-year-old, gravid 2, para 1 woman at 11 4/7 weeks of gestation was admitted to a local hospital because of fever and chest pain with breathing difficulty that had persisted for 1 day. Blood tests showed 8.24 × 10e9/L white blood cells and 148.7 mmol/L C-reactive protein. An ultrasound revealed left pleural effusion and a single live foetus in the uterus. A prophylactic antibiotic was initiated with ampicillin and azithromycin. Then, thoracic drainage was performed. Adenosine deaminase levels from the hydrothorax were found to be elevated to 58.20 U/L, and a blood T-SPOT was positive. An acid-fast TB bacillus stain obtained from the hydrothorax was positive, suggesting tuberculous pleurisy.

The TB regimen for tuberculous pleurisy is as below.

A first-line anti-TB drug regimen was initiated (INH at 0.3 g/day, RIF at 0.45 g/day, and PZA at 0.5 g/tid) for 10 days. Her chest pain was relieved. However, the patient had nausea with a fever of 38.1 °C, and her alanine transaminase (ALT) level reached 58 IU/L. The anti-TB treatment was stopped for 3 days due to possible hepatic toxicity. She was transferred to another municipal hospital. Her highest body temperature reached 40.4 °C, and the attending physician reinitiated the same anti-TB drugs for another 6 days. The jaundice of the patient became increasingly more apparent and her ALT level increased to 1325 IU/L. Total bilirubin was 44.8 µmol/L, and the prothrombin time (PT) was 39 s. All anti-TB drugs were discontinued.

The patient was transferred to our hospital. The patient was vomiting, she presented with jaundice, dark urine, and fatigue with normal vital signs at admission. The obstetrical examination showed an enlarged uterus without uterine activity or bleeding. Her laboratory work-up showed progressive hepatic failure (Table 1). In addition to some typical causes of hepatotoxicity, several pregnancy-related causes were excluded, such as acute fatty liver due to pregnancy, HELLP syndrome, and infection. The patient was denied contact with a known tuberculous patient and prohibited from consuming Chinese herbal medicines or alcohol. The patient married at 20 years old and had given birth to a healthy girl the previous year. Her personal and family medical history was unremarkable. According to the ultrasound scan, the liver bile ducts and hepatic vessels were normal. A multidisciplinary team of hepatologists, surgeons, physicians and obstetricians took care of the patient. An artificial liver support system (ALSS) was applied four times in combination with liver protection therapy for 10 days, but the patient’s clinical condition continued to decline. Her GCS score was 1 + 1 + 4 and her MELD score was 24. She was added to the super-urgent liver transplantation list. After graft allocation, an orthotopic LT was performed at 17 3/7 weeks of gestation. The operation time was 6 h 15 min, and the volume of blood loss was approximately 1000 mL with transfusions of 6 U of red blood cells. In our hospital, the second line anti-TB treatment before and after LT is initially amikacin at 0.4 g/day, levofloxacin at 0.4 g/day, and meropenem at 1 g q8 h by intravenous administration with the consent of the patient and her family members who were informed about the possible adverse drug effects on the foetus.

**Table 1:** Laboratory test values during inpatient admission (ALT, alanine transaminase; PT, prothrombin time; INR, international normalized ratio; WBC, white blood cell count; Hb, hemoglobin)

Laboratory	Admission day	Pre-op day 1	Day of LT	Post-op day 3	Post-op day 9
Date	08/01	08/09	08/10	08/12	08/18
Bilirubin in µmol/L (0–21)	172.3	283.2	235	200.2	66.1
Albumin in g/L (40–55)	33.7	37.7	39.4	37.8	36.2
ALT in U/L (7–40)	412	27	802	421	69
PT in s (10-13.5)	44.6	27.7	21.1	15.5	11.4
INR (0.85–1.15)	4.29	1.88	1.87	1.33	0.94
WBC*10e9/L (4–10)	9.3	20.3	17.7	15.9	10.2
Hb in g/L (113–151)	90	83	60	63	63
Serum creatinine in µmol/L (41–73)	26	30	33	29	32
Serum ammonia in µmol/L (10–47)	71	81	91	20	/

**Fig. 1:**
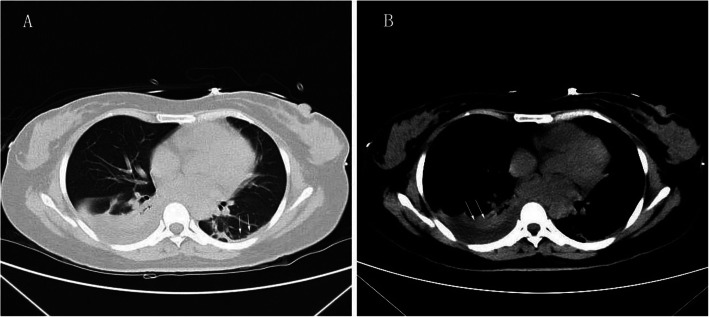
Lung CT scan showing left pleural thickening and right pleural effusion

**Fig. 2:**
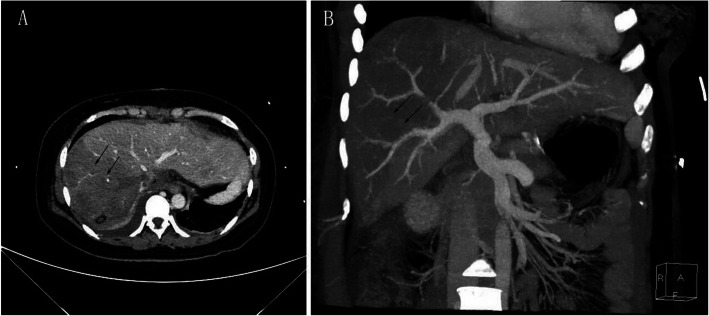
Liver angiography image showing blood vessels functioning with delayed right hepatic perfusion

**Fig. 3:**
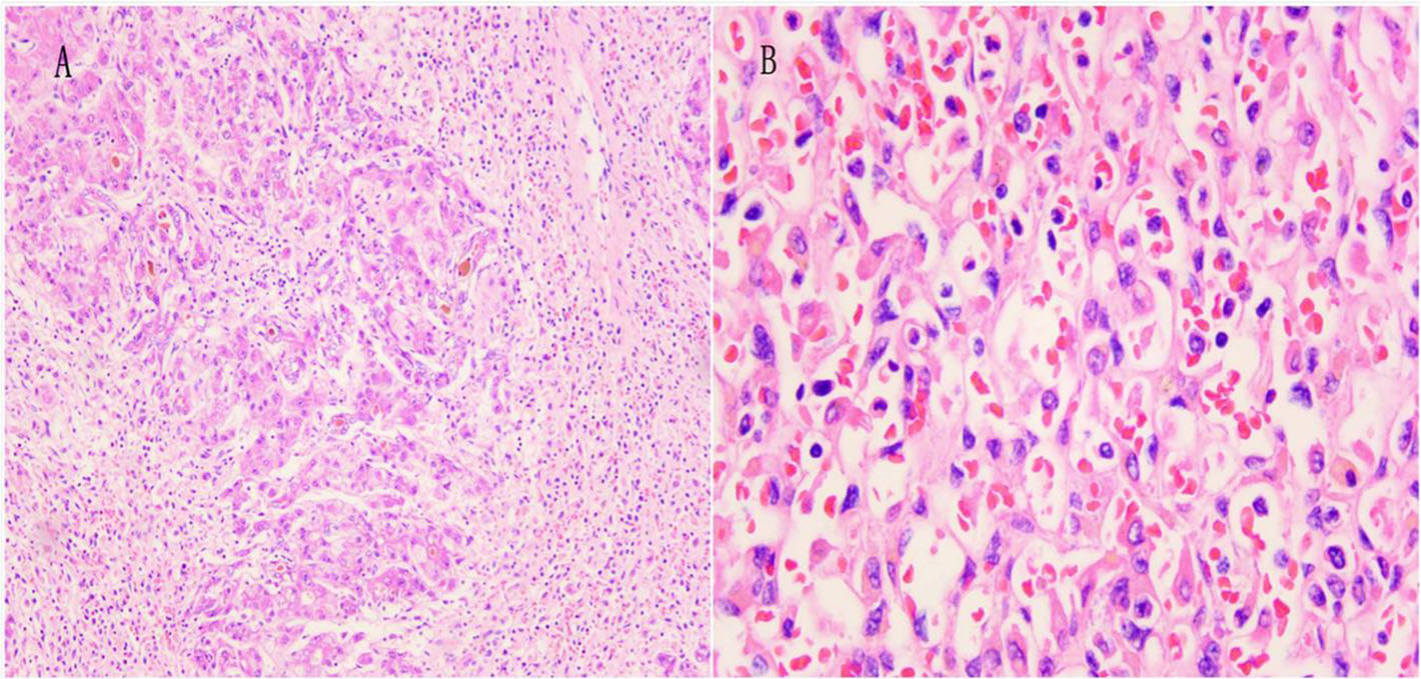
The histopathological examination showed sub-mass necrosis of the liver with cholestasis in liver cells

After LT, she received basiliximab and a methylprednisolone taper to induce immunosuppression; Mycophenolate sodium enteric-coated tablets, corticosteroids, tacrolimus for initial immunosuppression maintenance. The patient was extubated 18 h after surgery. On post-op day 5, a lung CT showed left pleural thickening and right pleural effusion (Fig. 1). On post-op day 6, no obvious improvements in her laboratory tests were evident, a liver angiography showed that the blood vessels were functioning but with delayed right hepatic perfusion (Fig. 2). Thus, low-molecular-weight heparin was used to anti-coagulate the blood. By post-op day 20, the patient’s allograft function had gradually improved. Then, the anti-TB regimen was changed to linezolid (LZD) at 0.6 g/day, levofloxacin at 500 mg/day, and pyridoxine at 100 mg/tid orally according to the recommendation of a TB expert. We modified the LZD according to blood concentrations. The histopathological examination showed submassive necrosis and cholestasis of the liver, which confirmed the diagnosis (Fig. 3). The foetus was managed by daily monitoring of the foetal heart rate. On post-op day 29, foetal sonography revealed mild bilateral ventricle widening, with the left side approximately 1.0 cm wide and the right side approximately 1.1 cm wide. The patient and her family decided to discontinue the pregnancy, providing ethical informed consent. Labour was induced via double balloon dilation for 12 h. On post-op day 30, the aborted foetus was vaginally delivered with spontaneous expulsion of the placenta, and the foetus had a normal appearance and weighed 280 g. A mother with active pulmonary TB can transmit the infection to her foetus, but the placental pathology of this patient was negative. The ultrasound scan indicated a possible incomplete abortion. On post-op day 37, we performed uterine curettage. The patient was then discharged. She has continued her anti-TB treatment and immunosuppression drugs as an outpatient. At the 3-month post-op follow-up, a routine chest CT revealed some ground-glass opacity nodules that turned out to be pulmonary aspergillosis, which responded to voriconazole (0.2 g/q12h for 14days). At the 8 months follow-up, the patient showed good general condition without TB relapse or liver damage.

## Discussion and conclusion

WHO recommends that active TB during pregnancy should be treated with four first-line drugs (rifampin, isoniazid, ethambutol and pyrazinamide) [8]. Of these, rifampin, isoniazid and pyrazinamide are known to induce hepatotoxicity [5]. Mild or fatal liver dysfunction is a major adverse effect, and it can cause treatment discontinuation or even death. Our hospital has reported that among 155 inpatients given anti-TB DILI, the overall mortality was 15 (9.68 %) from 2010 to 2016 [9]. The atypical symptoms of liver injury may be complicated by those of other pregnancy complications [10]. The careful observation of clinical symptoms such as anorexia, nausea, vomiting, dark urine, icterus, rash and close monitoring of liver function are essential for an early diagnosis. If any symptoms occur, the guidelines recommend discontinuing all drugs until the liver function tests become normal. The Task Force of the European Respiratory Society advises restarting all drugs simultaneously after a first episode of hepatotoxicity and reintroducing the drugs consecutively after a second episode of hepatotoxicity. The American Thoracic Society advises restarting anti-TB drugs one at a time [4]. Our case shows that we should be more prudent when anti-TB drugs are restarted in a pregnant patient. When severe liver injury occurs, ALSS can temporarily support a patients’ liver function, and improve their preoperative condition, thus extending the waiting time for a donor liver and serving as a bridge to LT [11]. It was used in our patient, and the waiting time for a donor liver was 10 days. Liver failure cannot be reversed, and liver transplantation is the inevitable choice in our patient. There is little worldwide experience with liver transplantation in pregnant patients, although many pregnancies with positive outcomes have been reported when LT was completed before the pregnancy [12]. A multidisciplinary team of hepatologists, surgeons, physicians and obstetricians discussed an optimal schedule for the patient. The challenge of LT in pregnancy is haemodynamic control and special consideration to avoid compression of the inferior vena cava by the pregnant uterus [13].

The timing of pregnancy termination and liver transplantation is a debatable topic. In a related study, 18 cases of LT between the 11th to 27th weeks of pregnancy have been reported with a prenatal mortality rate of 50 % [14]. Depending on the gestation week and the viability of the foetus, termination of the pregnancy has to be discussed with the patient. If the foetus is expected to survive, X-ray blocking equipment should be used to protect the foetus, and foetal toxic drugs such as mycophenolate mofetil should be avoided. In our study, therapeutic abortion was considered an option by the patient and her family, who provided ethical informed consent. As soon as the allograft function was stable, we induced labour. For a pregnancy in a patient with severe liver dysfunction, mechanical devices can be used to induce labour, such as double-balloon dilation [15], which is a quick and effective method of induction. Interestingly, foetal lateral ventriculomegaly has been reported in three previous cases of LT during pregnancy [14, 16, 17].

The treatment of TB after LT is more challenging due to drug toxicity and drug-drug interactions. In fact, RIF and INH induce the metabolism of immunosuppressive drugs such as cyclosporine, FK, and corticosteroids, via induction of the cytochrome P450 pathway. Consequently, the use of RIF is associated with a higher rate of rejection [18]. Since the hepatotoxic occurred in our patient, RIF, INH and PZA-sparing regimens are preferred. Despite several case reports, second-line therapy for TB has not yet been systematically studied in transplant recipients [19]. In a Spanish cohort of SOT patients with TB, quinolones and LZD were promising alternatives to INH and RIF [20]. Another review suggested that EMB and a fluoroquinolone may be safe and effective [7]. Second-line TB drugs may carry greater risks to both the mother and child, such as aminoglycosides, which are ototoxic and nephrotoxic for both the mother and the foetus. Quinolones have teratogenic potential and can cause skeletal deformities. Doctors should talk about the regimen with the patient and their families patiently. When our patient exhibited confusion or was comatose, we used second-line intravenous anti-TB drugs, including meropenem, amikacin and levofloxacin. After the patient recovered from the LT surgery, we used oral LZD, pyridoxine, and levofloxacin to maintain the treatment effects. We minimized the dosage of LZD upon therapeutic drug monitoring. The full treatment was resumed after the 8 months follow-up. There are several other non-TB drugs such as antihypertensive, antithyroid, and antibiotics that may cause drug-induced ALF in pregnancy that we should also be aware of [21–23].

In conclusion, our case report highlights the need to raise awareness about the possibility of liver failure during anti-TB treatment in pregnancy. An artificial liver support system combined with liver transplantation may be an option for these patients even though pregnant women may experience more complexed situation. Mechanical devices can be wise choices to induce labour for a pregnancy with liver dysfunction. A multidisciplinary approach is essential to optimize patient outcomes. To our best of knowledge, this is the first pregnant case of liver transplantation for FHF caused by anti-TB drugs. Further research is needed to identify risks of TB treatment and liver failure and liver transplantation in pregnancy women and foetuses.

## Data Availability

The datasets used during the current study are available from the corresponding author on reasonable request.

## Abbreviations

TB: Tuberculosis
FHF: Fulminant hepatic failure
ALSS: Artificial liver support system
DILI: Drug-induced liver injury
LT: Liver transplantation
ALT: Alanine transaminase
PT: Prothrombin time
ALSS: Artificial liver support system
LZD: Linezolid
